# Factors Affecting Fatigue among Nurses during the COVID-19 Pandemic

**DOI:** 10.3390/ijerph191811380

**Published:** 2022-09-09

**Authors:** Haeyoung Lee, Seunghye Choi

**Affiliations:** 1 Red Cross College of Nursing, Chung-Ang University, 84 Heukseok-ro, Dongjak-gu, Seoul 06974, Korea; 2 College of Nursing, Gachon University, 191, Hambangmoe-ro, Yeonsu-gu, Incheon 21936, Korea

**Keywords:** COVID-19, depression, fatigue, nurses, stress

## Abstract

This study identified clinical nurses’ fatigue and related factors during the COVID-19 pandemic. This was a cross-sectional study. Data were collected from South Korean hospitals on 234 nurses’ general characteristics, fatigue, depression, occupational stress, insomnia, and perceived daytime sleepiness using a structured questionnaire. The prevalence of fatigue was 62.0%, depression 52.1%, insomnia 20.7%, and daytime sleepiness 36.1%. Insomnia, sleepiness, depression, and occupational stress were significantly associated with fatigue. Ward nurses who cared for COVID-19 patients within the past month had significantly higher occupational stress related to organizational climate than those who had not provided care, and ICU nurses who cared for COVID-19 patients had significantly higher job insecurity-related occupational stress. Nurses have a high prevalence of fatigue and depression during the pandemic. Thus, insomnia, sleepiness, depression, and occupational stress must be reduced to lower nurses’ fatigue. Caring for COVID-19 patients was not significantly associated with fatigue, but there were significant differences in occupational stress between nurses who provided such care and those who did not. Work environment-specific strategies are needed to reduce nurses’ occupational stress during the pandemic.

## 1. Introduction

Fatigue is a mentally or physically exhausted state wrought by overwork, and it is an outcome of the interaction of various factors, such as physical factors of the modern industrial structure, environmental factors, and individuals’ psychological factors. Prolonged fatigue leads to deterioration of health, morbidity, and reduced productivity in workers [1]. Owing to the nature of their work, nurses must constantly acquire new knowledge and skills, and make important decisions related to human lives; this exposes them to a higher level of stress than people in other occupations [2]. A hospital setting in particular has heavy workloads and non-standard work schedules, causing a high level of physical and mental stress, in which case the incidence of fatigue may be higher [3,4].

Relieving nurses’ fatigue is critical for improving healthcare quality [5]. Nurses’ fatigue affects nurses’ health and work performance, their organizations, and their patients. Thus, nurses’ fatigue impacts medical malpractice and patient safety, reduces productivity, increases medical and social costs, and may lead to accidents and disease [6]. A longitudinal study found that nurses’ fatigue is significantly associated with turnover [7].

Previous studies have identified the predictors of nurses’ fatigue as physical factors (working on the computer, working on foot or moving or lifting patients, taking vital signs, preparing injections, administering injections, excessive paperwork, tests, surgery, and treatment), mental factors (continued mental labor, organizational environment, conflict with healthcare providers, responsibility, and increased hospital demands), and emotional factors (anxiety, conflict, frustration, responsibility, worries, concern, depression, and personality type) [8]. Furthermore, nurses’ fatigue results in physical symptoms, such as diminished functioning, energy loss, and complete exhaustion, as well as discomfort in various aspects [8]. A study on first-line nurses in Wuhan reported that daily working hours, frequency of working night shifts, and lifestyle factors predicted nurses’ fatigue [9].

The associated factors of fatigue among nurses exposed to challenges owing to a new disease differ from the general predictors of nurses’ fatigue. A study observed that the fatigue of nurses providing care for Middle East Respiratory Syndrome (MERS), a novel infection at the time, was higher than that of clinical nurses, and was influenced by physical factors related to the use of personal protective equipment (PPE) and emotional factors related to concerns about lack of specific care knowledge and getting infected [10]. For the coronavirus disease 2019 (COVID-19) pandemic too, the relevant literature has reported that an increased number of confirmed or suspected cases, a shortage of PPE, and media coverage are predictors of diverse psychological problems, such as depression, anxiety, and insomnia in the frontline staff [11,12]. An online survey revealed that a substantial number of healthcare workers experienced depression (50.4%), anxiety (44.6%), and insomnia (34.0%) during the COVID-19 pandemic [13]. A study on frontline staff also reported a high rate of anxiety (22.4%), depression (50.0%), and fatigue (73.7%) [14]. Researches on fatigue and related factors among nurses working in multiple centers during the COVID-19 pandemic are scarce. Therefore, this study aimed to investigate the differences in fatigue, depression, insomnia, sleepiness, and occupational stress according to the provision of nursing care for COVID-19 patients, as well as the associated factors of nurses’ fatigue induced by caring for COVID-19 patients, to propose safe and efficient nursing staff management.

## 2. Methods

This study conducted a descriptive investigation to identify clinical nurses’ fatigue and its associated factors during the COVID-19 pandemic. Data was collected on nurses’ general characteristics, fatigue, depression, occupational stress, insomnia, and perceived daytime sleepiness using a structured questionnaire between January—March 2021 from several South Korean hospitals.

### 2.1. Participants and Setting

The participants of this study comprised nurses from multiple hospitals (eight general hospitals with 300 beds or more) in Seoul (four), Gyeonggi (two), Incheon (one), and Chunbuk (one) in South Korea, who understood the intention and purpose of this study and consented to participation. Those who had worked for less than six months as clinical nurses were excluded from the study. The number of samples was calculated at 200 using G*power version 3.1.9.2 (Düsseldorf, Germany) with an effect size of 0.25, a significance level of 0.05, and a power of 0.95 [15]. Snowball sampling was used to recruit participants from among those who voluntarily consented to participate. Data were collected from 250 participants considering an approximate 80% response rate. After excluding participants with incomplete questionnaires (*n* = 14) and those who did not sign the informed consent form (*n* = 2), 234 participants were included in the final analysis. 

### 2.2. Data Collection

Data was collected using a structured questionnaire from January—March 2021. The study was approved by the relevant ethics committee. Data were collected after obtaining the permission and cooperation of the hospitals’ nursing departments. We visited the hospitals in person, explained the purpose and intention of the study, and collected self-reported surveys from consenting participants. The confidentiality and anonymity of the materials were explained, together with the study’s purpose and intention. Participants were also informed that they could refuse or discontinue participation at any time during the process. Those who were informed and consented to participate were asked to sign a written informed consent form. 

### 2.3. Measurements 

A structured questionnaire with 82 questions was used. Nurses’ general characteristics collected included sex, age, total length of employment in hospital (years), duration of career in current ward (years), current work unit (ward, ICU, OR, ER, outpatient, other), current position (acting nurse, charge nurse, unit manager, nurse practitioner, other), shift work (yes or no), living arrangement (live with family, dorm, live alone), marriage (yes or no), nursing care experience of COVID-19 within the last month (yes or no), illness within the last month (yes or no), used medication within the last month (yes or no), fatigue, depression, occupational stress, insomnia, and daytime sleepiness.

Fatigue was measured using the Fatigue Severity Scale (FSS) developed by Krupp, LaRocca, Muir-Nash, and Steinberg [16]. The FSS comprises nine items that assessed the level of fatigue in the past week on a scale from 1–7. The overall score is an average of the individual item scores and could range between 1–7. A higher mean score indicates a higher level of fatigue. FSS scores of four or higher are considered as fatigue [16]. Therefore, the researchers operationally defined a nurse with an FSS score of 4 or higher as a fatigue group and an FSS score of less than four as a non-fatigue group within this study. Cronbach’s alpha ranged from 0.81–0.89 in the previous study [16] and was 0.91 in this study.

Depression was measured using the Center for Epidemiologic Studies Short Depression Scale developed by Radloff [17]. This scale comprises 20 items with four factors: depressed affect, positive affect, somatic and retarded activity, and interpersonal relations [17]. The possible range for the 20-item scale is 0–60; a cutoff score of 16 or higher indicates the presence of significant depressive symptoms [18]. Cronbach’s alpha was 0.86 in the study conducted by Li and Hicks [19] and 0.91 in this study.

Occupational stress was measured using the Korean Occupational Stress Scale developed by Chang et al. [20]. This 26-item tool uses a four-point scale to measure eight categories: physical environment (two items), job demands (four items), insufficient job control (four items), interpersonal conflict (three items), job insecurity (two items), organizational system (four items), lack of reward (three items), and occupational climate (four items). As the number of items differs for each category, the score for each category was converted to a 100-point score to prevent an unbalanced estimation of the categories. The conversion equation is: converted score = (actual score-number of items) × 100/(highest anticipated score-number of items). The total job stress score is the total score for all eight categories divided by eight. The converted score ranges from 0–100; a higher score indicates a higher degree of job stress [20]. Cronbach’s alpha was 0.512–0.822 at the time of development [20] and 0.80 in this study.

The insomnia severity index (ISI) was used to assess sleep disorders [21]. It is reported to be appropriate for measuring sleep disorders in shift workers [22]. The ISI consists of seven items related to sleep disorders, each rated on a five-point scale (0–4). A total score (0–28) of 0–7 indicates no clinically significant insomnia, 8–14 indicates subthreshold insomnia, 15–21 indicates clinical insomnia (moderate severity), and 22–28 indicates clinical insomnia (severe). Cronbach’s alpha was 0.74 in the previous study [23] and 0.90 in this study.

Participants perceived daytime sleepiness was measured using the Epworth Sleepiness Scale [24]. This questionnaire-based scale is known to provide valid measurements of sleep propensity in adults [24]. It defines a score of below 10 as normal, and that of 10 or higher as daytime sleepiness [25]. Cronbach’s alpha was 0.88 in the study conducted by Johns and Hocking [25] and 0.85 in this study.

### 2.4. Ethical Considerations

Before commencement, this study protocol was approved by the Institutional Ethical Committee Review Board of Gachon University (IRB no: 1044396-202012-HR-206-01). Informed consent was confirmed by the IRB and obtained from each participant.

### 2.5. Data Analysis

Data were analyzed using IBM SPSS version 26. The threshold for statistical significance was set at *p* < 0.05 for all the analyses. Participants’ general characteristics were calculated using the real number, percentage, mean and standard deviation. A *t*-test and *χ*^2^ -test were conducted to identify the differences between the general characteristics of nurses, depression, insomnia, daytime sleepiness, and occupational stress according to fatigue. After the Kolmogorov–Smirnov normality test, a non-parametric Spearman correlation coefficient was used to determine the correlation between age, fatigue, and occupational stress. 

A multiple logistic regression analysis was conducted to examine the factors affecting nurses’ fatigue. The multiple logistic regression analysis included variables with significant differences in fatigue by univariate analysis (insomnia, sleepiness, depression, occupational stress, and illness). Prior to performing the multiple logistic regression, the Hosmer and Lemeshow was conducted to identify the goodness of fit. The model of this study could be considered reasonably good as *p* = 0.567. Additional analyses compared the relationships among insomnia, sleepiness, depression, and occupational stress according to the provision of care for COVID-19 patients. To eliminate the differences caused by the severity of COVID-19, separate analyses were performed for nurses who were then working in a patient ward and those who were then working in the ICU. *t*-tests were conducted to identify the differences in general characteristics, fatigue, depression, insomnia, sleepiness, and occupational stress according to the history of providing care for COVID-19 patients in the previous month. 

## 3. Results

### 3.1. Participants’ Characteristics

Of the 234 participants, 87.2% were women, and the mean age was 33.37 ± 8.34 years (Table 1). The mean total length of nurses’ employment in the hospital was 8.29 ± 7.80 years (Table 1). A total of 62.0% (*n* = 145) of the nurses experienced fatigue, and the total score of occupational stress was 46.14 ± 9.68 (Table 1). A total of 77.4% (*n* = 181) of the participants worked in rotating shifts, and 59.9% (*n* = 139) had provided care for COVID-19 patients within the previous month (Table 2). A total of 52.1% (*n* = 122) suffered depression. A total of 20.7% (*n* = 48) of the nurses had clinical insomnia (moderate-severe), and 36.1% (*n* = 84) had daytime sleepiness (Table 2).

### 3.2. Participants’ General Characteristics, Depression, Insomnia, Daytime Sleepiness, and Occupational Stress

The rate of experienced illness within the previous month and depression were higher in the fatigue group than in the non-fatigue group (*p* < 0.014 and *p* < 0.001, respectively). Total insomnia and the rate of clinical insomnia were higher in the fatigue group than in the non-fatigue group (*p* < 0.001 and *p* < 0.001, respectively). Moreover, daytime sleepiness, total occupational stress, physical environment (occupational stress factor 1), job demand (occupational stress factor 2), interpersonal conflict (occupational stress factor 4), organizational system (occupational stress factor 6), lack of reward (occupational stress factor 7), and occupational climate (occupational stress factor 8) were higher in the fatigue group than in the non-fatigue group (*p* < 0.001, *p* < 0.001, *p* = 0.006, *p* < 0.001, *p* = 0.009, *p* = 0.035, *p* = 0.001, *p* = 0.011, respectively; Table 2).

### 3.3. Correlation between Fatigue, Age, and Occupational Stress

Fatigue was positively correlated with total occupational stress, physical environment, job demand, interpersonal conflict, organizational system, lack of reward, and occupational climate (*r* = 0.346, *p* < 0.001; *r* = 0.281, *p* < 0.001; *r* = 0.316, *p* < 0.001, *r* = 0.198, *p* = 0.002; *r* = 0.180, *p* = 0.006; *r* = 0.242, *p* < 0.001; and *r* = 0.171, *p* = 0.009, respectively; Table 3). 

### 3.4. Associated Factors of Fatigue

For the logistic regression model, we entered the associated variables that were related to fatigue. Furthermore, regression results revealed that insomnia (*p* = 0.014), sleepiness (*p* = 0.037), depression (*p* = 0.005), and job demand (occupational stress factor 2; *p* = 0.022) were significantly associated with fatigue (Table 4). These associated factors accounted for 33.1% of the variance in fatigue (Table 4).

### 3.5. Differences in Fatigue, Depression, Insomnia, Sleepiness, and Occupational Stress according to Provision of Nursing Care for COVID-19 Patients

Additional analyses were performed to examine the relationship between providing care for COVID-19 patients with fatigue, depression, insomnia, sleepiness, and occupational stress. Among nurses who were then working in a patient ward, those who had provided care for COVID-19 patients in the previous month had a significantly longer total length of employment in the hospital but a significantly shorter duration of a career in the current ward (*p* = 0.036, *p* = 0.001) (Table 5). Furthermore, nurses who had provided care for COVID-19 patients within the past month had significantly lower job demand factors of occupational stress and significantly higher occupational climate factor stress than those who had not provided care (*p* = 0.034, *p* = 0.001) (Table 5).

Among nurses who were then working in the ICU, those who had provided care for COVID-19 patients in the previous month had a significantly lower job demand factor of occupational stress but a significantly higher job insecurity factor stress than those who had not provided care (*p* < 0.001, *p* = 0.001).

## 4. Discussion

Nurses experience unstable psychological reactions as they faced infectious diseases with a highly contagious virus in the context of a pandemic [26]. In this study, the prevalence of fatigue was 62%, and the mean insomnia score was 9.68 ± 5.77. A previous study in South Korea using the same tool reported a fatigue rate of 60.7–78.6% [27], while a study of ICU nurses working rotating shifts using the same tool reported a mean insomnia score of 11.30 ± 6.47 [22]. Thus, compared with the previous studies conducted in Korea, our participants had a similar rate of fatigue and a lower level of insomnia. These results may be due to the differences in the participants’ current work unit. The participants of this study were working in various hospitals and departments including wards and outpatient. However, a previous study in South Korea was conducted for a special unit in a single hospital [27]. However, the degree of fatigue and insomnia of the participants in the present study was higher than that of nurses in other countries. A study among Polish nurses showed that the prevalence of fatigue of 35.3–50.5% and mean insomnia score (7.87 ± 4.42) were lower than those revealed by the results of this study [28].

Approximately 52.1% of the participants of this study had depression, and 20.7% experienced clinical insomnia. A previous study reported that 50.4% and 34.0% of healthcare workers experienced depression and insomnia, respectively, during the COVID-19 pandemic [13] and that 50.0% of frontline staff experienced depression [14]. Therefore, we inferred that nurses experienced severe depression during the COVID-19 pandemic.

According to the results of this study, insomnia, sleepiness, depression, and occupational stress were associated with nurses’ fatigue. Fatigue is a multidimensional symptom, and fatigue and depression are highly comorbid [29]. Insomnia is a key indicator of co-occurring fatigue and depression [29]. Occupational stress is a major health problem for both employees and organizations, and could lead to burnout, illness, and employee turnover [30]. According to a previous study, frontline nurses experienced high levels of stress and burnout, and moderate depression during the COVID-19 pandemic [31]. Our participants were primarily nurses working in the metropolitan area, which had the highest incidence of COVID-19 in South Korea. Although the level of occupational stress among these participants was not markedly high, preventive interventions in mental health are needed for nurses who provide care for COVID-19 patients, as mental health problems, such as depression, and stress, significantly influence nurses’ physical and mental fatigue [9].

Providing care for COVID-19 patients was not significantly associated with nurses’ fatigue. The relationship between COVID-19 patients cares and nurse fatigue was inconsistent even in the previous studies [14,32]. This may be related to the experience of nurses who cared for quarantined patients. According to a previous study conducted in Korea, the nurses who cared for COVID-19 patients experienced exhaustion; however, at the same time, they adapted and felt pride [26]. The sources to boost morale were cooperation with colleagues and support from the manager. In the regression model, job demands were related to fatigue among nurses. However, both ward and ICU nurses who provided care for COVID-19 patients within the past month had significantly lower job demands than the other group. The items included in the job demand factor were time pressure, responsibility, and burden from workload [20]. Therefore, if systematic support is provided to the nurses, providing nursing care for COVID-19 itself would not be a factor that increases nurses’ fatigue. Moreover, it may be related to South Korea’s relatively low COVID-19 mortality rate (1.67%) compared with other countries [33].

In this study, ward nurses and ICU nurses differed in their scores for the subscales of occupational stress. Ward nurses who provided care for COVID-19 patients within the previous month had significantly higher occupational climate factors among occupational stress. Meanwhile, ICU nurses who provided care for COVID-19 patients within the past month had significantly higher job insecurity factors among occupational stress. The items included in the occupational climate factor were “given work orders with no standard and consistency; workplace climate is authoritative and vertical”, and the items included in the job insecurity factor were “future is uncertain, and undesirable changes in work conditions or situations are anticipated [20]”. Approximately 43.3% of the participants of this study were then working in a ward, most of whom did not experience the care of isolated patients as a part of their routine work. In Korea, as the need for medical personnel rapidly increased, nurses who did not have adequate training for high contagiousness patients were assigned to COVID-19 nursing, or the working department was suddenly changed. Such a sudden change in working environment could lead to psychological distress [26]. Therefore, nursing care for COVID-19 patients might not be a simple job transition, especially for ward nurses. It could be a factor increasing occupational climate factor-related stress. Compared with the previous findings, nurses demonstrated a low job insecurity factor, as they are professionals with relatively high job security [34]; however, the participants of this study who provided care for severe COVID-19 patients in ICU exhibited high job insecurity factor related to occupational stress. Job insecurity and occupational climate factors among occupational stress were significantly correlated with depression [34]. The results of this study also revealed that occupational stress and depression were significantly correlated. This calls for strategies to reduce occupational stress. A previous study showed that ethical leadership is a critical buffering factor for the negative influence of job insecurity in an organization [35]. Thus, nursing organizations that treat COVID-19 patients need to promote work unit-specific leadership that reduces occupational climate-related stress and job insecurity stress.

The level of occupational stress of the participants in this study was not markedly increased compared to the previous study that explored nurses’ occupational stress and fatigue before the COVID-19 pandemic [27]. However, there was a difference in the order among the subscale of occupational stress before the COVID-19 pandemic, and in the context of the COVID-19 pandemic. Before the COVID-19 pandemic, occupational stress was found to be high in the order of job demand and lack of reward [27]. However, in this study, it was found to be high in the order of the job demand and physical environment. During the COVID-19 pandemic, nurses experienced high physical environment-related occupational stress. Physical environmental-related occupational stress may be due to heavy workload [36]. According to a previous study, the main stressors of the nurses were fear of social isolation, discomfort due to protective equipment, and the burden on the patient [37]. The nurses taking care of the COVID-19 patients experienced stress owing to the uncertain nature of COVID-19 and inadequate protection such as personal protective equipment (PPE), which can complicate COVID-19 management [38]. Providing safety physical environment such as sufficient PPE to nurses caring for COVID-19 patients is highly important for the safety of patients and nurses, so nursing managers should ensure adequate PPE supply [39]. This could have a positive effect on reducing the occupational stress of nurses caring for COVID-19 patients.

### Limitations

This study has the following limitations. First, it was a cross-sectional study; thus, we could not draw conclusions about the causality between nurses’ fatigue and the factors associated with it. Second, we relied on self-reported data for nurses’ fatigue and insomnia. Subsequent studies should objectively measure fatigue and insomnia, such as by using physiological parameters, and examining the association between them. Third, nurses’ work-related characteristics, such as daily working hours and frequency of working night shifts could not be considered, even though these factors influence fatigue. This study was conducted in hospitals with a quarantine ward for COVID-19 patients; therefore, the number of questions was limited to minimize the time consumed to respond to the questionnaire. Future studies should objectively measure and include various work-related characteristics, such as actual working hours and frequency of working night shifts. Fourth, the vast majority of the participants were female nurses who worked in a hospital in the Seoul metropolitan region. Future studies should be conducted with a more diverse study population to accumulate data.

## 5. Conclusions

This study found that nurses have experienced a high level of fatigue and depression during the COVID-19 pandemic. Insomnia, sleepiness, depression, and occupational stress were associated with nurses’ fatigue. While providing care for COVID-19 patients itself was not significantly associated with fatigue, there were significant differences in occupational stress between nurses who provided care for COVID-19 patients and those who did not. Thus, insomnia, sleepiness, depression, and occupational stress must be alleviated to lower nurses’ fatigue, and the job insecurity factor and occupational climate factor of occupational stress should be improved to diminish occupational stress in nurses assigned to COVID-19 patient care.

## Figures and Tables

**Table 1:** Participants Characteristics (N = 234).

Variable	N (%) or M ± SD	Possible Score Range
Male	30 (12.8)	
Female	204 (87.2)	
Age (years)	33.37 ± 8.34	
Total length of employment in hospital (years)	8.29 ± 7.80	
Duration of career in current ward (years)	2.76 ± 4.03	
Fatigue	4.27 ± 1.09	1–7
Yes	145 (62.0)	
No	89 (38.0)	
Depression	17.74 ± 9.99	0–60
Occupational stress	46.14 ± 9.68	0–100
Factor 1	57.12 ± 23.04	0–100
Factor 2	59.83 ± 18.29	0–100
Factor 3	48.21 ± 13.75	0–100
Factor 4	31.43 ± 13.27	0–100
Factor 5	37.91 ± 27.21	0–100
Factor 6	49.36 ± 16.11	0–100
Factor 7	47.35 ± 16.30	0–100
Factor 8	38.13 ± 17.85	0–100
Insomnia	9.68 ± 5.77	0–28
Daytime sleepiness	8.40 ± 4.65	0–24

Note-SD, standard deviation; ICU, intensive care unit; OR, operating room; ER, emergency room; Factor 1, physical environment; Factor 2, job demand; Factor 3, insufficient job control; Factor 4, interpersonal conflict; Factor 5, job insecurity; Factor 6, organizational system; Factor 7, lack of reward; Factor 8, occupational climate.

**Table 2:** Participants’ general characteristics, depression, insomnia, daytime sleepiness, and occupational stress (N = 234).

Variable	Category	Total N(%)	Fatigue Group N(%) or M ± SD	Non-Fatigue Group N(%) or M ± SD	*χ*^2^ or *t*	*p*
Gender	Female	204 (87.2)	126 (86.9)	78 (87.6)	0.027	0.519
Age			33.28 ± 7.91	33.51 ± 9.05	0.202	0.840
Current work unit	Ward	101 (43.3)	63 (43.8)	38 (42.7)	10.386	0.065
	ICU	57 (24.5)	31 (21.5)	26 (29.2)		
	OR	22 (9.4)	10 (6.9)	12 (13.5)		
	ER	7 (3.0)	5 (3.5)	2 (2.2)		
	Outpatient	23 (9.9)	20 (13.9)	3 (3.4)		
	Other	23 (9.9)	15 (10.4)	8 (9.0)		
Current position	Acting nurse	195 (83.3)	119 (82.1)	76 (85.4)	2.959	0.565
	Charge nurse	22 (9.4)	15 (10.3)	7 (7.9)		
	Unit manager	10 (4.3)	5 (3.4)	5 (5.6)		
	Nurse practitioner	2 (0.9)	2 (1.4)	0 (0.0)		
	Other	5 (1.7)	4 (2.8)	1 (1.1)		
Shift worker	Yes	181 (77.4)	107 (73.8)	74 (83.1)	2.754	0.066
Living arrangement	Live with family	131 (56.0)	85 (58.6)	46 (51.7)	2.907	0.408
	Dorm	25 (10.7)	16 (11.0)	9 (10.1)		
	Live alone	77 (32.9)	44 (30.3)	33 (37.1)		
Marriage	Yes	91 (39.4)	59 (41.0)	31 (35.6)	1.327	0.515
Nursing care experience of COVID-19 within the last month	Yes	139 (59.9)	81 (56.3)	58 (65.9)	2.122	0.093
Experienced illness within the last month	Yes	21 (9.0)	18 (12.5)	3 (3.4)	5.483	0.014
Used medication within the last month	Yes	61 (26.4)	42 (29.2)	19 (21.8)	1.498	0.142
Depression *	Yes	122 (52.1)	96 (66.2)	26 (29.2)	30.244	<0.001
Insomnia	Total		11.23 ± 5.66	7.14 ± 5.01	−5.576	<0.001
	No clinically significant insomnia	90 (38.8)	41 (28.5)	49 (55.7)	22.761	<0.001
	Subthreshold insomnia	94 (40.5)	62 (43.1)	32 (36.4)		
	Clinical insomnia, moderate severity	42 (18.1)	35 (24.3)	7 (8.0)		
	Clinical insomnia, severe	6 (2.6)	6 (4.2)	0 (0.0)		
Daytime sleepiness	Total		9.25 ± 4.49	7.02 ± 4.61	−3.643	<0.001
	Normal (no daytime sleepiness)	149 (63.9)	80 (55.6)	69 (77.5)	11.519	<0.001
	Have daytime sleepiness	84 (36.1)	64 (44.4)	20 (22.5)		
Occupational stress	Total		48.24 ± 9.03	42.78 ± 9.79	−4.314	<0.001
	Factor 1		60.35 ± 21.43	51.87 ± 24.67	−2.770	0.006
	Factor 2		63.39 ± 17.69	54.03 ± 17.86	−3.917	<0.001
	Factor 3		48.09 ± 13.65	48.41 ± 13.98	0.171	0.864
	Factor 4		33.18 ± 13.42	28.54 ± 12.58	−2.622	0.009
	Factor 5		39.24 ± 27.42	35.77 ± 26.89	−0.945	0.346
	Factor 6		51.10 ± 16.29	46.54 ± 15.48	−2.117	0.035
	Factor 7		50.00 ± 16.36	43.07 ± 15.34	−3.216	0.001
	Factor 8		40.45 ± 16.49	34.36 ± 19.37	−2.559	0.011

Note-SD, standard deviation; ICU, intensive care unit; OR, operating room; ER, emergency room; *, Depression was measured using the Center for Epidemiologic Studies Short Depression Scale, and the cut-off score was 16; Factor 1, physical environment; Factor 2, job demand; Factor 3, insufficient job control; Factor 4, interpersonal conflict; Factor 5, job insecurity; Factor 6, organizational system; Factor 7, lack of reward; Factor 8, occupational climate.

**Table 3:** Correlation between fatigue, age, and occupational stress (N = 234).

Variable	Fatigue	Age	Occupational Stress
	*r* (*p*)	*r* (*p*)	*r* (*p*)
Fatigue	1	0.029 (0.662)	0.0346 (<0.001)
Age	0.029 (0.662)	1	−0.095 (0.154)
Occupational stress (total)	0.346 (<0.001)	−0.095 (0.154)	1
Factor 1	0.281 (<.001)	−0.100 (0.130)	0.600 (<0.001)
Factor 2	0.316 (<0.001)	−0.037 (0.576)	0.488 (<0.001)
Factor 3	−0.044 (0.500)	−0.192 (0.003)	0.311 (<0.001)
Factor 4	0.198 (0.002)	0.123 (0.061)	0.416 (<0.001)
Factor 5	0.059 (0.371)	−0.128 (0.052)	0.330 (<0.001)
Factor 6	0.180 (0.006)	0.027 (0.678)	0.651 (<0.001)
Factor 7	0.242 (<0.001)	−0.061 (0.356)	0.680 (<0.001)
Factor 8	0.171 (0.009)	0.052 (0.430)	0.616 (<0.001)

Factor 1, physical environment; Factor 2, job demand; Factor 3, insufficient job control; Factor 4, interpersonal conflict; Factor 5, job insecurity; Factor 6, organizational system; Factor 7, lack of reward; Factor 8, occupational climate.

**Table 4:** Associated factors of fatigue (N = 234).

Independent Variable	*B*	*SE*	*p*	Adjusted OR	95% CI	
					Lower	Upper
(Constant)	−3.315	0.935	<0.001	0.036		
Insomnia	0.085	0.035	0.014	1.089	1.018	1.165
Sleepiness	0.094	0.045	0.037	1.098	1.006	1.199
Depression (yes)	0.991	0.350	0.005	2.694	1.356	5.352
Occupational stress (total)	0.008	0.038	0.836	1.008	0.936	1.085
Factor 2	0.025	0.011	0.022	1.025	1.003	1.047
Factor 4	0.023	0.015	0.115	1.023	0.994	1.053
Factor 6	−0.015	0.015	0.312	0.985	0.957	1.014
Factor 7	−0.004	0.015	0.799	0.996	0.968	1.025
Factor 8	0.003	0.012	0.821	1.003	0.979	1.027
Current illness (yes)	1.304	0.710	0.066	3.684	0.916	14.820
Nagelkerke R^2^ = 0.331						

Note-SE, standard error; OR, odds ratio; CI, confidence interval; Factor 2, job demand; Factor 4, interpersonal conflict; Factor 6, organizational system; Factor 7, lack of reward; Factor 8, occupational climate.

**Table 5:** General characteristics, fatigue, depression, insomnia, sleepiness, and occupational stress according to the history of providing care for COVID-19 patients (N = 146).

	Nurses Who Work in a Patient Ward (N = 90)				Nurses Who Work in ICU (N = 56)			
Variables	Yes (N = 71) M ± SD	No (N = 29) M ± SD	*t*	*p*	Yes (N = 45) M ± SD	No (N = 11) M ± SD	*t*	*p*
Age	33.03 ± 8.13	29.59 ± 7.64	2.006	0.050	32.07 ± 7.30	29.55 ± 6.33	1.050	0.298
Total length of employment in hospital (years)	7.26 ± 7.77	4.62 ± 4.45	2.132	0.036	6.58 ± 6.45	6.19 ± 6.77	0.177	0.860
Duration of career in current ward (years)	1.33 ± 1.55	3.01 ± 2.44	−3.452	0.001	1.33 ± 3.50	3.45 ± 3.95	−1.761	0.084
Fatigue	4.15 ± 1.10	4.49 ± 1.11	−1.401	0.164	4.14 ± 1.18	3.99 ± 1.11	0.373	0.711
Depression	19.07 ± 11.47	19.21 ± 10.68	−0.055	0.956	16.91 ± 9.45	17.18 ± 10.25	−0.084	0.933
Insomnia	9.51 ± 5.80	10.64 ± 5.76	−0.872	0.385	9.49 ± 5.96	9.36 ± 7.57	0.059	0.953
Sleepiness	8.26 ± 5.54	9.00 ± 4.69	−0.633	0.528	8.00 ± 4.15	9.73 ± 4.86	−1.197	0.237
Occupational stress	47.47 ± 10.60	44.51 ± 9.16	1.297	0.198	44.79 ± 8.30	43.21 ± 5.29	0.599	0.552
Factor 1	58.69 ± 22.69	51.72 ± 27.22	1.312	0.192	58.89 ± 22.92	59.09 ± 13.67	−0.028	0.978
Factor 2	57.51 ± 16.50	65.80 ± 19.84	−2.148	0.034	51.48 ± 19.65	65.91 ± 5.84	−4.222	<0.001
Factor 3	47.89 ± 14.89	48.85 ± 10.38	−0.318	0.751	44.81 ± 13.09	42.42 ± 13.67	0.538	0.592
Factor 4	32.06 ± 13.71	28.74 ± 15.00	1.069	0.288	28.89 ± 13.48	30.30 ± 12.26	−0.317	0.753
Factor 5	42.49 ± 24.53	38.10 ± 26.00	0.789	0.432	52.22 ± 34.92	27.27 ± 15.41	3.576	0.001
Factor 6	49.65 ± 16.99	48.85 ± 16.63	0.214	0.831	45.93 ± 13.95	46.21 ± 16.40	−0.059	0.953
Factor 7	48.57 ± 17.15	45.98 ± 17.25	0.684	0.496	42.96 ± 14.13	40.40 ± 15.13	0.531	0.598
Factor 8	43.45 ± 19.24	29.60 ± 18.04	3.319	0.001	33.15 ± 17.27	34.09 ± 13.67	−0.168	0.867

Note-SD, standard deviation; ICU, intensive care unit; Factor 1, physical environment; Factor 2, job demand; Factor 3, insufficient job control; Factor 4, interpersonal conflict; Factor 5, job insecurity; Factor 6, organizational system; Factor 7, lack of reward; Factor 8, occupational climate.

## Data Availability

The datasets generated and/or analyzed during the current study are not publicly available to protect the participants but are available from the corresponding author on reasonable request.
